# 3T-MRI Artificial Intelligence in Patients with Invasive Breast Cancer to Predict Distant Metastasis Status: A Pilot Study

**DOI:** 10.3390/cancers15010036

**Published:** 2022-12-21

**Authors:** Alessandro Calabrese, Domiziana Santucci, Michela Gravina, Eliodoro Faiella, Ermanno Cordelli, Paolo Soda, Giulio Iannello, Carlo Sansone, Bruno Beomonte Zobel, Carlo Catalano, Carlo de Felice

**Affiliations:** 1 Department of Radiology, University of Rome “Sapienza”, Viale del Policlinico 155, 00161 Roma, Italy; 2 Department of Radiology, Sant’Anna Hospital, Via Ravona, 22042 San Fermo della Battaglia, Italy; 3 Unit of Computer Systems and Bioinformatics, Department of Engineering, University of Rome “Campus Bio-Medico”, Via Alvaro del Portillo 21, 00128 Roma, Italy; 4 Department of Electrical Engineering and Information Technology, University of Naples Federico II, 80131 Naples, Italy; 5 Department of Radiation Sciences, Radiation Physics, Biomedical Engineering, Umeå University, Universitetstorget, 490187 Umeå, Sweden; 6 Department of Radiology, University of Rome “Campus Bio-medico”, Via Alvaro del Portillo, 21, 00128 Rome, Italy

**Keywords:** breast cancer, 3T-MRI Dynamic Contrast-Enhanced sequences (DCE), Deep Learning (DL), convolution neural network (CNN), metastasis

## Abstract

**Simple Summary:**

Breast cancer is still the most common cancer in the female population and is the second leading cause of cancer death in women. Although only 6% of breast cancers have metastatic spread at onset, metastases remain the first cause of death. An artificial intelligence approach could be a valuable noninvasive predictor of the risk of distant metastasis. The purpose of this study is to determine the role of a Deep Learning model approach based on a convolutional neural network in predicting the risk of distant metastasis in patients with breast cancer using dynamic Contrast-Enhanced 3T-MRI images.

**Abstract:**

Background: The incidence of breast cancer metastasis has decreased over the years. However, 20–30% of patients with early breast cancer still die from metastases. The purpose of this study is to evaluate the performance of a Deep Learning Convolutional Neural Networks (CNN) model to predict the risk of distant metastasis using 3T-MRI DCE sequences (Dynamic Contrast-Enhanced). Methods: A total of 157 breast cancer patients who underwent staging 3T-MRI examinations from January 2011 to July 2022 were retrospectively examined. Patient data, tumor histological and MRI characteristics, and clinical and imaging follow-up examinations of up to 7 years were collected. Of the 157 MRI examinations, 39/157 patients (40 lesions) had distant metastases, while 118/157 patients (120 lesions) were negative for distant metastases (control group). We analyzed the role of the Deep Learning technique using a single variable size bounding box (SVB) option and employed a Voxel Based (VB) NET CNN model. The CNN performance was evaluated in terms of accuracy, sensitivity, specificity, and area under the ROC curve (AUC). Results: The VB-NET model obtained a sensitivity, specificity, accuracy, and AUC of 52.50%, 80.51%, 73.42%, and 68.56%, respectively. A significant correlation was found between the risk of distant metastasis and tumor size, and the expression of PgR and HER2. Conclusions: We demonstrated a currently insufficient ability of the Deep Learning approach in predicting a distant metastasis status in patients with BC using CNNs.

## 1. Introduction

Currently, patients with early invasive breast cancer (BC) are mainly treated by surgical approaches, with or without radiotherapy, possibly preceded by neoadjuvant chemotherapy and followed by adjuvant systemic therapy to reduce the risk of recurrence and distant metastasis. BC presenting with distant metastasis at onset corresponds to about less than 6% of all invasive BC [1,2].

Few studies exist that investigate the incidences of metastasis in patients with BC and the relative mean time to metastasis onset and the subsequent survival. A large study from the German cancer registry described that the proportion of patients without metastases at diagnosis, but who later developed metastases within 5 years of initial diagnosis, decreased from 27% in 1978–1984 to 15% in 1995–2003. The same study showed a reduction in the proportion of bone metastases, with a relative increase in metastases to other sites, mainly the liver and brain [3].

The risk of occurrence of distant metastases can be influenced by different factors such as tumor stage, grade, and subtype. A large Australian study demonstrated a difference in the 5-year risk of metastasis between women with node-negative disease (5.3%) and those with node-positive or locally advanced disease at diagnosis (18.1%) [4].

To ensure the best treatment and achieve the best results, accurate BC staging is essential. Magnetic Resonance Imaging (MRI) has demonstrated itself to be a highly sensitive and non-invasive technique that provides both morphologic and functional data. Currently, MRI is used in the diagnosis step, preoperative tumor staging, and evaluation of response to chemotherapy treatment [5]. Most of the tumor information on MRI is qualitative and radiologist-dependent. Despite this, Apparent Diffusion Coefficient (ADC) values were found to correlate with both grading and biopsy, and surgical cellularity [6,7].

In recent years, image-based Machine Learning (ML) processes have been employed in many oncological fields. Radiomics is a relatively new area of artificial intelligence (AI), first developed by Lambin et al. [8]. It involves extracting and converting a large number of quantitative data (called “features”) from medical images. After careful selection, ML models are trained to provide prediction tools for various outcomes. When combined with image-derived clinical and qualitative analyses, these data can aid in medical decision making, potentially improving patient prognosis and lesion characterization.

AI aims to assist physicians in evaluating lesions beyond the subjective visual interpretation that is possible with currently employed methodologies. Although it is still a developing area of research, the effects of radiomics-derived data analysis on lesion diagnosis, prediction of response to chemotherapy, risk of recurrence, disease-free survival, axillary lymph node status, and the influence of tumor edema in histologic characterization of BC have already been studied in the field of BC [9,10,11]. However, considerable study heterogeneity has been found in the literature, mainly regarding the specifics of the radiomic approach [10].

Building a ML system requires engineering and expertise to design an extractor that transforms the features into an appropriate representation that the learning subsystem can detect or classify. For this reason, there has been an emergence of the Deep Learning (DL) approach, which improves this process by automatically discovering the representations needed for the specific task from the raw data [12]. A key role is played by Convolutional Neural Networks (CNNs), which are a set of deep architectures that receive raw image data as input and extract features to learn discriminative properties using hierarchical representations of images.

Deep Learning has already been applied to BC-MRI in the lesion diagnosis, prediction of tumor molecular subtypes, pathological complete response, and axillary lymph node status [13,14,15,16,17]. To our knowledge, there are no articles that have studied the role of AI through DL in predicting the risk of distant metastasis from primary BC. Therefore, the purpose of this study is to develop a DL-CNN model to predict the risk of distant metastasis using Contrast-Enhanced 3T-MRI.

## 2. Materials and Methods

### 2.1. Study Population

This is a retrospective observational study where only existing information collected from human participants was used, with no identifiers linking individuals to the data/samples. All methods and procedures meet institutional and research committee ethical standards in accordance with the 2013 Declaration of Helsinki.

All breast 3T-MRI exams performed at the Radiology Department of our hospital, from January 2011 to July 2022 for pre-operative evaluation, were retrospectively reviewed. The inclusion criteria were as follows: (a) pre-operative breast 3T-MRI with Dynamic DCE-MRI sequences; (b) diagnostic confirmation of invasive breast cancer by histopathological analysis; (c) complete histological analysis including molecular receptor status (estrogen receptor ER; progesterone receptor PgR; epidermal growth factor receptor HER2) and proliferation index Ki-67; (d) patients undergoing clinical and imaging follow-up at our institution for at least seven years or until distant metastases diagnosis.

The presence of breast implants, post-chemotherapy follow-up patients, exams for neo-adjuvant treatment evaluation, and images that were not of good diagnostic quality were all considered as exclusion criteria.

Written informed consent was obtained for all patients before MRI.

Following the mentioned criteria, a total of 157 breast cancer patients with 160 breast lesions were included in the study. Patients clinical data (age, menopausal state, family history, hormone therapy) were collected.

### 2.2. MRI Imaging and Data Analysis

All MRI exams were performed on a 3T magnet (Discovery 750; GE Healthcare, Milwaukee, WI, USA). Patients were positioned prone and a dedicated eight-channel breast coil (8US TORSOPA) was used. Three orthogonal localizer sequences were employed, then images were acquired following this protocol:

- T2-weighted axial single-shot fast spin echo sequence with a modified Dixon technique (IDEAL) for intravoxel fat-water separation (TR/TE 3500–5200/120–135 ms, slice thickness 3.5 mm).
- Diffusion-weighted axial single-shot echo-planar with fat suppression sequence.
- (TR/TE 2700/58 ms, slice thickness 5 mm) with diffusion-sensitizing gradient with a *b*-value of 0, 500, and 1000 s/mm^2^.
- Dynamic 3D-T1w axial and sagittal gradient echo sequence with fat suppression after injection of 0.1 mmol/kg body weight of Gadoteric acid (Dotarem^®^, Guerbet S.p.A., Villepinte France, or Claricyclic^®^, GE Healthcare S.r.l, Chicago, IL, USA) at a rate of 2 mL/sec followed by a bolus of 15 mL saline flush (TR/TE 4/2 ms, slice thickness 2.4 mm), before, and five to ten times after intravenous contrast medium injection.

Subtracted images were automatically produced in post-processing from the images after contrast medium administration for a more accurate tumor analysis. The largest lesion was considered to be the index lesion for statistical analysis purposes. Reading was performed without access to the original reports and clinical data.

The following MRI characteristics were collected for each lesion using DCE sequences as reference images for tumor detection and characterization.

- Location on the breast quadrant;
- Margins: regular, irregular, lobulated, speculated, non-mass;
- Size (mm);
- Morphology: round, oval, or irregular.

For each index lesion, a signal-intensity-to-time curve (SI/T) was automatically generated by placing a region of interest (ROI) within the lesion on a subjectively recognized area of maximal contrast enhancement and evaluating all the acquired DCE series. The kinetics curves were classified as I (progressive wash-in), II (plateau) or III (rapid wash-out), as reported in the current ACR BI-RADS guidelines [18].

ADC values were calculated for quantitative analysis by superimposing the subtracted images on the ADC map. An ROI with a diameter of 3–6 mm was manually drawn on the slice where the lesion reached its greatest diameter. ADC measurements were performed only on the enhanced solid portion to avoid areas of T2 shine-through.

Distant metastasis status for each patient was recorded using clinical and imaging follow-up, with definitive histological characterization confirming breast cancer cells as a dichotomous result: positive, if there was at least one organ with metastases, or negative, if there were no metastases. Radiological examinations (MRI, CT, and PET) and medical records of the Patients were collected up to a maximum of seven years after the initial diagnosis of breast cancer; if distant metastases were found, their organ site was recorded.

### 2.3. Histologic Characteristics

All breast lesions were characterized on the histological specimen obtained by a core biopsy and on the histological definitive sample after surgery by two pathologists. Histological diagnosis was performed according to the WHO classification. 

The histopathological grade was evaluated according to the Nottingham Grading System, considering tubule formation, pleomorphism, and mitotic count through a scoring system. The total score ranges from 3 to 9: 3–5 corresponds to grade 1 (G1), 6 or 7 to grade 2 (G2), and 8 or 9 to grade 3 (G3).

Immunohistochemical (IHC) analysis was performed to evaluate molecular receptor status (ER, PgR, and HER2) and to calculate the Ki-67 index. Evaluation of ER and PgR status was performed by IHC using Dako monoclonal antibody, 1:100 dilution. The monoclonal antibody Mib-1 (1:200 dilution; Dako, Glostrup, Denmark) was used to assess the Ki-67 index, which was reported as the percentage of immune-reactive cells out of 2000 tumor cells in randomly selected high-power fields surrounding the tumor core. HER2 status was re-evaluated using the Hercep test (Dako, Glostrup, Denmark), following published guidelines. Samples that gave an equivocal IHC result were subjected to fluorescence in situ hybridization (FISH) analysis. A ratio of HER2 gene signals to chromosome 17 signals greater than 2.2 was used as a cut-off value to define HER2 gene amplification. ER and PgR status were considered to be positive if the expression was >1% and negative if the expression was <1%. HER2 expression was classified as 0, 1+, 2+ or 3+; only tumors reaching a score of 3+ were considered to be HER2-positive.

### 2.4. Segmentation and Pre-Processing

Each case was anonymized and identified with a progressive identification number (ID).

For the analysis of bilateral tumors, lesions were considered one at a time with different IDs. Image preparation was performed on a personal workstation using 3D Slicer (version 5.0, The Slicer Community, Brigham and Women’s Hospital, Boston, USA): a freely available open-source software. For each tumor, the second post-contrast subtracted T1w sequence was selected. For each case, a label map was generated. Using manual and thresholding-assisted segmentation techniques, the lesions were drawn manually. Segmentation was initially always performed in the axial projections and subsequently reshaped and optimized in the other projections until an optimal lesion contour was obtained. Multifocal or multicentric lesions were also segmented.

### 2.5. Volumes Extraction

**Figure 1:**
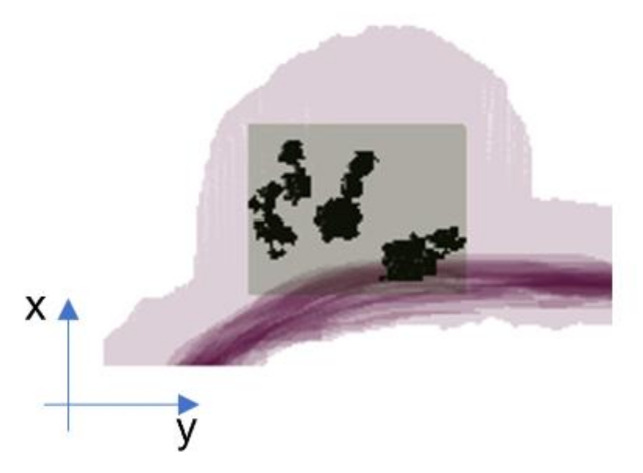
Representation of the SVB option, only the smallest 3D bounding box confined to the tumor region is considered.

To limit the amount of non-tumor tissue to be included, the smallest 3D rectangle circumscribed by the tumor region was considered for each patient in Single Variable-size Box (SVB), as shown in Figure 1. 

Denoting with dxp × dyp × dzp the size of the bounding rectangle of patient p, a 3D cubic box of size dmp × dmp × dmp is used, where dmp = max(dxp, dyp, dzp), to crop the subject. The cubic box of the SVB option depends on the patient. Therefore, the amount of non-tumor tissue depends on the shape of each patient’s tumor region and the difference between the largest (dmp) and maximum (dxp, dyp, dzp) size, and the difference between the largest (dmp) and smallest (dmp) size.

However, in the case of multifocal and multicentric tumors, the parenchyma between lesions was included in the 4D volume extracted. Each voxel was associated with its size information in millimeters (mm). During the acquisition of the DCE sequence for patient p, the Pixel Spacing attribute was the physical distance between the centers of each two-dimensional pixel, specified by two numerical values (ˆxp, ˆyp) that represented the row spacing and vertical spacing, respectively. In addition, the attribute spacing between slices specified by the numerical value ˆzp was the distance between slices measured along the normal to the first image. 

### 2.6. Metastasis Prevision Assessment

We exploited CNNs for the prediction of extra-nodal metastasis. The voxel-based (VB)-NET model was used, considering that the size of the selection rectangle varies according to the tumor region of each patient.

The proposed networks consist of several reduction layers and two fully connected layers. A reduction layer is a block with a convolutional layer, followed by a normalization layer and the ReLU function. Each convolutional layer reduces the dimensionality of the input feature map and doubles the number of channels, making pooling operations (maximum or average pooling) unnecessary.

**Figure 2:**
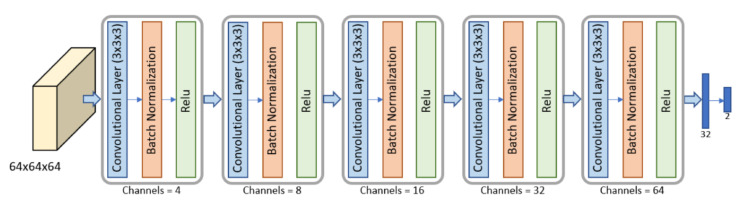
Architecture of the VB-NET used for the SVB. The VB-NET is a 3D CNN with five reduction blocks and two fully connected layers.

The architecture of VB-NET is shown in Figure 2. We used a 3D CNN, consisting of five reduction blocks. The input volume (4D data) represented the smallest cubic box surrounding the tumor region or lesion of each patient. Because the SVB considered a selection rectangle size that varies according to each patient’s tumor region, resizing was used to give the volumes a common size of 64 × 64 × 64, before feeding them to the VB-NET.

Performance was evaluated in terms of accuracy, sensitivity, specificity, and area under the ROC curve (AUC). The experiment was performed in a 10-fold cross-validation (CV) setting. A patient-based cross-validation was performed to reliably estimate performance, avoiding the use of 3D volumes or slices of the same patient during the training and evaluation phase. The experiment was conducted using Pytorch (version 1.10, Meta AI, Astor Place, New York, NY, USA), while the pre-processing phase, including the different bounding box options, were implemented in MATLAB 2020b.

### 2.7. Statistical Analysis

Descriptive statistics were carried out using the statistical software SPSS© version 25.0. Statistical significance was set at *p* < 0.05. Spearman’s rank-order correlation was evaluated to assess whether there was a correlation between the presence of distant metastases during follow-up and categorical variables, clinical (menopausal status, hormone therapy, family history), MRI (site, margins, size, morphology, kinetic curves), and histologic features (histologic type, grading, expression of ER, PgR and HER2). The Kolmogorov–Smirnov test was performed to determine whether age, tumor size, Ki-67 index, and ADC values followed a normal distribution.

Statistical comparisons between the presence of distant metastases during follow-up and age, tumor size, Ki-67 index, and ADC values were performed using the Kruskal–Wallis H test.

## 3. Results

In this study, 157 breast cancer patients with 160 lesions were included. Three patients had bilateral breast cancer, but only one patient with bilateral BC presented distant metastases at follow-up. The mean age of the patients was 55 years (range 30–85 years). The patients were divided into two groups:

- Patients with distant metastases at follow-up (39/157 patients, 40 lesions);
- Patients negative for distant metastasis (control group, 118/157 patients, 120 lesions).

Metastasis sites are represented as follows: 3 (5%) brain, 17 (31%) lung, 19 (34%) bone, 14 (25%) liver, 1 (2%) gluteal subcutis, and 1 (2%) brachial plexus.

Anamnestic and clinical data of the study population were collected. The mean age of patients with metastases at follow-up was 54.3 years (range 30–84 years), while the mean age of the control group was 55.2 years (range 30–85 years). The average time to occurrence of distant metastasis was 22.8 months (range: 1–84 months).

The mean diameters of the measured lesions in the study population, the group with distant metastases, and the control group were 22.6 mm (range 6–90 mm), 33.3 mm (range 7–90 mm), 19.7 mm (range 6–80 mm), respectively.

**Figure 3:**
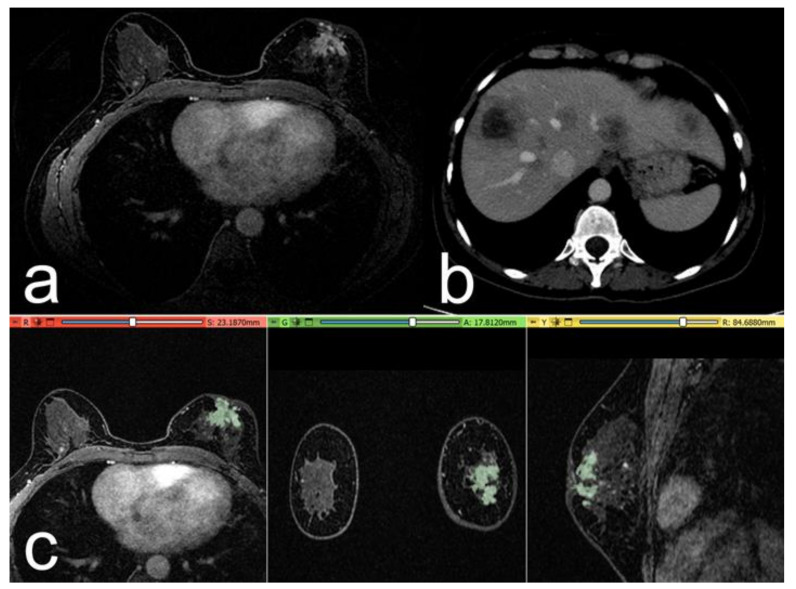
Case of a 52-year-old woman with a G3 triple negative invasive ductal carcinoma, Ki-67 of 30%. (**a**) The post-contrast image shows a retroareolar irregular non-mass-enhancing lesion in the left breast. (**b**) At 1-year follow-up: post-contrast axial CT image, with some metastatic nodules in the liver parenchyma. (**c**) Representation of the extraction of the segmentation mask.

**Figure 4:**
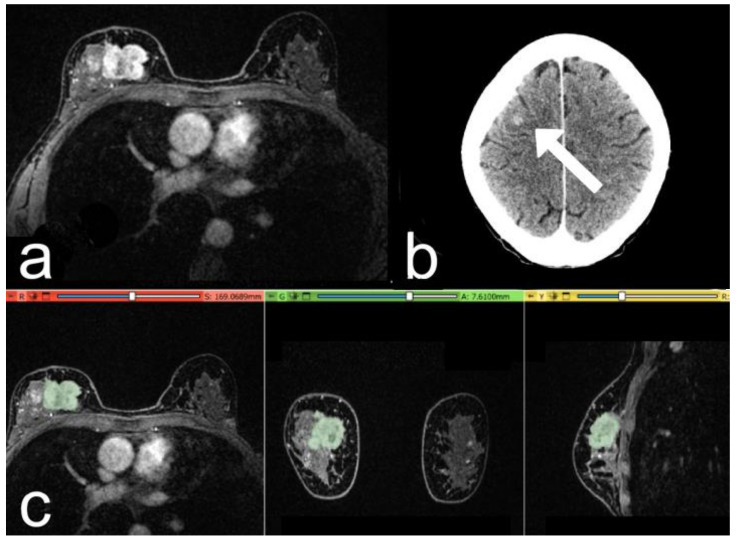
Case of a 53-year-old woman with a G2 Luminal B invasive ductal carcinoma, Ki-67 of 25%. (**a**) The post-contrast image shows an irregular mass-enhancing lesion in the upper quadrants of the right breast. (**b**) At 3-year follow-up: post-contrast axial CT image, with a metastatic nodule in the right frontal lobe (arrow). (**c**) Representation of the extraction of the segmentation mask.

The main imaging, histological, and clinical characteristics of the patients are shown in Table 1, Table 2 and Table 3. Two examples are reported in Figure 3 and Figure 4.

Using Spearman’s rank test, no correlation was found between the presence of distant metastasis at follow-up and menopausal status, family history, hormone therapy, lesion margins and morphology, kinetic curve, histologic type, histologic class, grading, and ER expression (*p* > 0.5). A significant correlation was found between the presence of distant metastasis at follow-up and the expression of PgR and HER2. The Kruskal–Wallis H test demonstrated a significant correlation between the presence of distant metastasis at follow-up and lesion size (*p* value < 0.001). No correlation was found between the presence of distant metastasis at follow-up, patient age, ADC values, and Ki-67 index.

The VB-NET model achieved a sensitivity, specificity, accuracy, and AUC of 52.50%, 80.51%, 73.42%, and 68.56%, respectively.

## 4. Discussion

Conflicting evidence exists regarding the improved survival of metastatic patients from BC. Some studies have found that the development and spread of adjuvant systemic therapies have resulted in a reduction in the spread of BC metastases, but not an increase in survival, due to the shift from lesions developing in sites with higher survival rates, such as the bone, to more aggressive sites such as the brain and liver [3]. However, according to other studies [19,20,21,22,23], adjuvant therapy has increased patient survival.

BC can be characterized by clinical cancer dormancy [24,25], and a wide window of relapse, which can range from months to decades after treatment of the primary tumor. This is related to the characteristic histologic heterogeneity of breast tumors; due to a higher incidence rate of metastasis, basal-like, and HER2-positive BC having a worse prognosis than tumors that express the estrogen receptor [26]. Therefore, in order to further individualize BC treatment for each patient, it is crucial to identify a potential risk marker for metastasis development.

AI has the ability to infer data from the images that are not visible to the subjective eye of the radiologist. Our aim was to test in our sample whether using a 3T-MRI-based Deep Learning model we can non-invasively predict the risk of distant metastasis from BC. We also studied whether clinical, MRI, or histologic features could play a similar role in this task. 

Ours is the first study that attempts to build a predictive model based on CNN Deep Learning for predicting distant metastasis of BC. However, our results demonstrated low AUC, accuracy, and sensitivity values of 68.56%, 80.51%, and 52.50%, respectively. These results reflect a currently insufficient capacity of the Deep Learning approach with CNN in its ability to predict the development of distant metastasis. In our previous studies, we have shown that both a radiomic and a DL approach can predict with greater than 80% accuracy the lymph node status of patients with BC [16,27]. We assume that this is due to the presence of lymphovascular invasion phenomena, some of which are not perceptible to the human eye. In the radiomic approach with a convex hull optimized segmentation, and in DL with the SVB bounding option, mammary regions adjacent to the tumor were also analyzed, obtaining information on possible lymphatic spread. For the same reason, we found that radiomics applied to the study of peritumoral edema also contribute significantly to predicting tumor histology and prognosis [11].

Furthermore, in this study no correlation was found between the risk of distant metastasis and almost all MRI, histologic, and clinical features considered. Tumor size, and therefore tumor stage, have a very good correlation with the risk of metastasis, as already found in the literature [28,29]; however, Sopik et al. determined that there is a linear relationship between metastatic risk and primary tumor size only for lesions between 7 and 60 mm, while both very small and very large tumors do not respect a linear relationship. No correlation was found between ADC values and the risk of metastasis; on the contrary, Kim et al. found that patients with lower minimum ADC values and higher ADC difference values had worse distant metastasis-free survival [30]. Tumors with these characteristics had greater cellularity and intratumoral heterogeneity, corresponding to greater aggressiveness.

With a DL approach, which is more complex than conventional radiomics, we did not find a meaningful performance of the model for several possible factors. BC is a heterogeneous disease, characterized by considerable pathophysiologic and histologic variability: the different molecular subtypes each have a different rate of aggressiveness and a different mean period free of recurrence or relapse. In addition, each molecular subtype has preferential sites of metastasis development. In a study by Xiao et al., HR+ BCs were correlated with the risk of bone metastasis, HER2+ subtypes were significantly associated with higher rates of liver, brain, and lung metastasis, and triple-negative tumors had a higher rate of brain metastasis but a significantly lower rate of bone metastasis [31]. These differences imply variability in the risk of metastasis as well as their clinical manifestation. This necessarily implies that surgical and pharmacological treatment must be particularly tailored to the patient, an element that may lead to further variation in the risk of distant disease and the time required for the onset of metastasis. A recent study by Cheng et al. investigated and validated the radiomic approach for predicting the risk of metastasis from BC: in this study, patients with the same molecular subtype (triple negative BCs) and with metastasis only in the brain site were selected. We expect that achieving similar homogeneity of the selected patient cohort may result in increased performance of the AI model. 

Our study has some limits: this is a retrospective study, with a relatively small number of patients with highly heterogeneous BC types. In addition, we could not explore the possible differences between the different histologic and molecular subtypes, nor the risks of developing metastases for each organ site, due to the small sample size. Segmentation was performed by one radiologist using the same methodology for all lesions, but with a manual assisted approach, which is a method that is time-consuming as well as not error-free. Only the second post-contrast phase was used as the segmentation mask, which, although it is recognized as the phase with higher contrast resolution for BCs, could cause the loss of additional information available from the other sequences. In particular, by considering only the second post-contrast phase, we lost information on the temporal dimension of the post-contrast tumoral enhancement progression, which was instead included in our previous DL study on the prediction of loco-regional lymph node status, which might explain the higher accuracy obtained [16].

Therefore, several steps are needed to improve the current performance of our model: enrollment of more patients to alleviate histological differences in BCs, sharing of different datasets among multiple institutions, development of prospective and multicenter studies, and validation and comparison of additional AI models. Thus, the study population should be as homogeneous as possible, with equal histological and molecular subtypes, and metastases in the same sites. In this regard, in this study we found a significant correlation between HER2, PgR expression, and metastasis risk, so the first successive step should be to select a cohort of patients with the expression of these two specific molecular receptors.

## 5. Conclusions

Our study demonstrated a currently insufficient ability of the Deep Learning approach in predicting distant metastasis status in patients with BC using CNNs. Our results suggest that further studies are necessary to investigate the role of AI in this specific task.

## Figures and Tables

**Table 1:** Description of the extracted MRI characteristics.

Variation			Study Population	Patients with Metastasis	Control Group	*p-*Value
Kinetic Curve	I	*n*	21	7	14	
		%	13.1%	17.5%	11.7%	
	II	*n*	71	15	56	
						0.962
		%	44.4%	37.5%	46.7%	
	III	*n*	68	18	50	
		%	42.5%	45.0%	41.7%	
Margins	Regular	*n*	4	0	4	
		%	2.5%	0.0%	3.3%	
	Irregular	*n*	86	21	65	
		%	53.8%	52.5%	54.2%	
	Lobulated	*n*	25	7	18	0.349
		%	15.6%	17.5%	15.0%	
	Spiculated	*n*	33	6	27	
		%	20.6%	15.0%	22.5%	
	Non-mass	*n*	12	6	6	
		%	7.5%	15.0%	5.0%	

**Table 2:** Description of the extracted histologic characteristics. * indicates statistical significance (*p* < 0.05).

Variation			Study Population	Patients with Metastasis	Control Group	*p-*Value
Histology	IDC	*n*	127	30	97	
		%	79.4%	75.0%	80.8%	
	ILC	*n*	33	10	23	0.433
		%	20.6%	25.0%	19.2%	
Molecular subtype	Luminal A	*n*	59	11	48	
		%	36.9%	27.5%	40.0%	
	Luminal B	*n*	69	18	51	
		%	43.1%	45.0%	42.5%	
	HER2+	*n*	13	3	10	0.079
		%	8.1%	7.5%	8.3%	
	Triple negative	*n*	19	8	11	
			11.9%	20.0%	9.2%	
ER Status	Negative	*n*	31	12	19	
		%	19.4%	30.0%	15.8%	
	Positive	*n*	129	28	101	0.195
		%	80.6%	70.0%	84.2%	
PgR Status	Negative	*n*	58	23	35	
		%	36.3%	57.5%	29.2%	
	Positive	*n*	102	17	85	0.001 *
		%	63.7%	42.5%	70.8%	
HER2 Status	Negative	*n*	137	32	105	
		%	85.6%	80.0%	87.5%	
	Positive	*n*	23	8	15	0.044 *
			14.4%	20.0%	12.5%	
Grade	1	*n*	19	4	15	
		%	11.9%	10.0%	12.5%	
	2	*n*	71	15	56	0.225
		%	44.4%	37.5%	46.7%	
	3	*n*	70	21	49	
		%	43.8%	52.5%	40.8%	

IDC: Invasive Ductal Cancer; ILC: Invasive Lobular Cancer.

**Table 3:** Description of the extracted clinical characteristics.

Variation			Study Population	Patients with Metastasis	Control Group	*p-*Value
Menopause	Pre-	*n*	71	17	54	
		%	44.4%	42.5%	45.0%	
	Post-	*n*	89	23	66	0.784
		%	55.6%	57.5%	55.0%	
Hormone Therapy	None	*n*	109	39	35	
		%	90.8%	97.5%	29.2%	
	Positive	*n*	11	1	85	0.168
		%	9.2%	2.5%	70.8%	
Family History	No relatives	*n*	118	32	86	
		%	73.8%	80.0%	71.7%	
	≥1 relative with BC	*n*	42	8	34	0.303
			26.3%	20.0%	28.3%	

## Data Availability

Data available on request.
